# Protein–Polysaccharide Gel Systems for Antioxidant and Antimicrobial Delivery in Sustainable Food Packaging: A Review

**DOI:** 10.3390/gels12040297

**Published:** 2026-04-01

**Authors:** Dimitrie Stoica, Cezar-Ionuț Bichescu, Mariana-Carmelia Bălănică-Dragomir, Maricica Stoica, Mariana Stuparu-Crețu

**Affiliations:** 1 Department of Applied Sciences, Cross-Border Faculty, “Dunarea de Jos” University of Galati, 111 Domneasca Street, 800201 Galati, Romaniacarmelia.dragomir@ugal.ro (M.-C.B.-D.); 2 Department of Business Administration, Faculty of Economics and Business Administration, “Dunarea de Jos” University of Galati, 59-61 Balcescu Street, 800001 Galati, Romania; 3 Department of Life Sciences, Cross-Border Faculty, “Dunarea de Jos” University of Galati, 111 Domneasca Street, 800201 Galati, Romania; 4 Faculty of Medicine and Pharmacy, “Dunarea de Jos” University of Galati, 35 Alexandru Ioan Cuza Street, 800010 Galati, Romania

**Keywords:** protein–polysaccharide gels, controlled release, biopolymer hydrogels, active food systems, antimicrobial and antioxidant delivery, sustainable packaging, bioactive compounds

## Abstract

Global demand for sustainable food packaging materials has intensified research on bio-based biopolymer systems capable of delivering functional compounds. Among these, protein–polysaccharide gels have emerged as versatile matrices for the incorporation and controlled release of antioxidant and antimicrobial agents. This review examines recent advances in the design and functionality of protein–polysaccharide gel systems for active food packaging applications. Particular attention is given to representative hybrid matrices such as casein/chitosan, gelatin/alginate, and whey protein/pectin systems, highlighting their gelation mechanisms, molecular interactions, and physicochemical properties. Furthermore, the review explores the potential of agro-industrial and marine by-products as renewable sources of proteins, polysaccharides, and bioactive compounds within circular bioeconomy strategies. Current limitations related to stability, scalability, and regulatory compliance are also addressed. By integrating structural, functional, and sustainability perspectives, this work provides a comprehensive framework for the development of next-generation protein–polysaccharide gel carriers for active food packaging.

## 1. Introduction

Globalization has considerably increased access to diverse food products from any part of the world, intensifying the need to preserve authentic sensory attributes such as appearance, aroma, flavor, and texture throughout food supply chains [1]. At the same time, the growing complexity and geographic extension of modern food chains have increased product exposure to environmental, microbial, and oxidative deterioration, making spoilage control an even more pressing challenge. Food spoilage remains a major global concern, contributing substantially to food losses and waste while also raising important economic, environmental, and food security issues. These pressures reinforce the need for preservation strategies capable of delaying deterioration while maintaining food quality and safety. Conventional methods, including refrigeration, freezing, drying, fermentation, thermal processing, and chemical preservation, have long been used to extend shelf life and reduce spoilage. Within this broader preservation framework, packaging represents an essential complementary preservation tool, supporting the protection, storage, handling, and transportation of foods while helping to reduce losses across the supply chain [2,3]. In this context, petroleum-based packaging materials play a significant role in protecting food against physical, chemical, and biological damage, thereby helping to maintain safety and quality during storage [4,5,6,7,8,9]. However, the large-scale dependence on fossil-derived plastics has generated substantial environmental concerns. In 2022, approximately 149 million metric tons of plastic were used globally for packaging manufacturing, and this value is projected to nearly triple by 2060 [10]. The environmental burden associated with plastic production and disposal, including greenhouse gas emissions, energy-intensive manufacturing, and the accumulation of persistent plastic waste and microplastics, has become a critical sustainability challenge [11,12,13]. In response to these pressures and in line with increasing regulatory restrictions on plastic use and microplastic emissions, the transition toward sustainable, bio-based packaging systems has become imperative [14]. Among emerging alternatives, bio-based biopolymers are promising candidates due to their renewability, biodegradability, recyclability, and low carbon footprint. Representative examples include proteins and polysaccharides such as casein, whey proteins, gelatin, chitosan, alginate, and pectin, all of which have attracted significant attention as versatile macromolecules capable of forming three-dimensional gel networks. Unlike conventional passive packaging, which mainly acts as an inert barrier, gel-based biopolymer systems offer dynamic structural architectures that can be functionalized with antimicrobial or antioxidant compounds. As a result, they can actively contribute to maintaining food quality and extending shelf-life, rather than serving solely as a barrier to the external environment [6,9,15,16,17,18,19]. Their intrinsic gelation behavior, together with crosslinking density and network topology, supports not only mechanical reinforcement and barrier functionality but also the controlled incorporation and release of antioxidant and antimicrobial compounds. Active packaging systems intentionally integrate functional agents, such as polyphenols, essential oils, or omega-3 fatty acids, within the packaging matrix, headspace, or surface. In such systems, the packaging material, food product, and surrounding microenvironment interact synergistically. Gel-based protein–polysaccharide matrices are particularly attractive in this respect because their molecular architecture governs diffusion pathways, retention efficiency, and release kinetics of embedded bioactives. Consequently, understanding the physicochemical mechanisms underlying gel formation and interpolymeric interactions is essential for the design of next-generation active packaging materials [8,20,21,22,23,24,25,26,27]. Recent high-impact literature has substantially advanced the field of sustainable and active food packaging. Nevertheless, discussion of protein–polysaccharide gel systems remains fragmented across material, mechanistic, and application-oriented perspectives. Broad reviews have mainly focused on sustainability, functional additives, hydrogel development, and general packaging applications, thus providing valuable overviews of current progress while paying less attention to the comparative mechanistic interpretation of hybrid gel systems as structure-dependent delivery platforms [28,29,30]. More specialized studies have addressed protein–polysaccharide composites, complexes, conjugates, gelation processes, and bioactive delivery, offering important insights into intermolecular interactions, assembly pathways, and functional performance. However, these aspects are often examined separately rather than within an integrated framework linking gelation mechanisms, network architecture, and release behavior [31,32,33,34,35]. In parallel, research on controlled-release packaging has progressed considerably, particularly in the development of intelligent antimicrobial systems and stimuli-responsive delivery strategies, yet such studies rarely examine representative protein–polysaccharide gel matrices as comparable mechanistic platforms for active food packaging [36]. Likewise, recent investigations of specific systems, such as chitosan/casein and whey protein/pectin matrices, have generated valuable mechanistic evidence; however, these findings remain formulation-specific and have not been comparatively synthesized across representative hybrid systems [37,38]. Consequently, the field still lacks a critical and integrative perspective capable of explaining how gel network formation and intermolecular organization govern structure–function relationships and the controlled release of antioxidant and antimicrobial agents in protein–polysaccharide gel systems intended for food packaging. Against this background, the present review moves beyond isolated discussions of composition, functionality, or application by critically examining how gelation mechanisms and protein–polysaccharide interactions shape the structural and functional behavior of hybrid gel systems for active food packaging. Despite extensive research on single-component protein- or polysaccharide-based films, current protein–polysaccharide gel systems still present important limitations. In particular, the relationships among interpolymeric interactions, gel network architecture, mechanical strength, barrier performance, environmental stability, and controlled bioactive release remain insufficiently understood, especially under realistic storage and application conditions. Moreover, many available studies focus on individual formulations or isolated functional properties, without providing an integrated structure–function perspective that supports the rational design of hybrid systems for food packaging applications [30,39,40,41,42]. This need for a more predictive, structure-oriented design strategy is further supported by recent work on multicomponent printable food gels, in which the combined influence of proteins and hydrocolloids on rheology, structural fidelity, and gel strengthening was associated with non-covalent interactions such as hydrogen bonding and electrostatic effects, while experimental findings were complemented by computational analysis [43]. These limitations hinder the development of protein–polysaccharide gels with predictable performance under realistic storage and application conditions [40,44,45]. In this context, hybrid protein–polysaccharide gels emerge as promising complementary systems capable of overcoming the intrinsic limitations of mono-component matrices. Several recent reviews have addressed biodegradable polymers, active packaging materials, and biopolymer-based films, often emphasizing raw material sources, processing methods, sustainability aspects, or general functional performance in food packaging applications [46,47]. Reviews focused on hydrogels and protein/polysaccharide-based systems have also summarized preparation strategies, cross-linking mechanisms, film-forming approaches, and bioactive delivery potential [30,33]. However, fewer studies have systematically linked intermolecular interactions, gel network organization, and functional performance within an integrated structure-function perspective.

In this context, the present review focuses specifically on protein–polysaccharide gel carriers and ternary systems as multifunctional matrices for antioxidant and antimicrobial delivery. Rather than considering these materials mainly at the compositional or formulation level, this review integrates intermolecular interactions, gelation mechanisms, network architecture, mechanical and barrier properties, and controlled release behavior into a comparative structure–function–application framework. By highlighting recurrent trade-offs, current limitations, and scale-up challenges, it aims to provide a more predictive and design-oriented perspective for the development of next-generation active food packaging systems.

## 2. Gel-Forming Biopolymers for Food Packaging Applications

Naturally derived biopolymers, including proteins, polysaccharides, lipids, and nucleic acids, have attracted considerable attention as sustainable alternatives to petroleum-based packaging materials. Among these, proteins and polysaccharides are the most technologically relevant classes for gel-based food packaging due to their biodegradability, renewability, intrinsic gelation ability, film-forming capacity, and compatibility with a broad range of bioactive compounds [30,34,39,47,48,49,50,51]. Protein- and polysaccharide-based systems differ fundamentally in molecular architecture and gelation behavior. Protein-based biopolymers are valued for their cohesive network formation, mechanical strength, and gas barrier properties. Their gelation behavior is governed by conformational transitions, intermolecular aggregation driven by hydrophobic interactions, hydrogen bonding, and covalent disulfide linkages, as well as environmental triggers such as pH, temperature, and ionic strength [39,47,50]. In contrast, polysaccharides, the most abundant biopolymers in nature, form networks predominantly through ionic crosslinking, hydrogen bonding, and electrostatic interactions between charged functional groups, with gel formation strongly influenced by charge density, degree of esterification, and ion diffusion dynamics [51,52]. Although numerous protein- and polysaccharide-based biopolymers have been investigated for packaging applications, this review focuses on casein, whey proteins, gelatin, chitosan, alginate, and pectin. These materials are among the most extensively studied gel-forming biopolymers in active and biodegradable packaging and represent complementary mechanistic classes relevant to hybrid gel formation and bioactive delivery [30,39,53,54]. They also form well-documented binary systems that provide a suitable basis for discussing gelation behavior, interpolymeric interactions, and structure–function–release relationships [39,40,53,54,55,56,57,58,59,60,61,62,63,64,65]. Beyond their structural role, these biopolymers can serve as carrier matrices for antioxidant and antimicrobial agents, thereby contributing to improved food safety and quality [39,48]. Other biopolymers, including starch, cellulose derivatives, and carrageenan, were not discussed here in order to maintain a focused review scope centered on representative protein–polysaccharide gel systems [30,39,53,54].

### 2.1. Renewable Sources and Valorization Pathways

**Figure 1:**
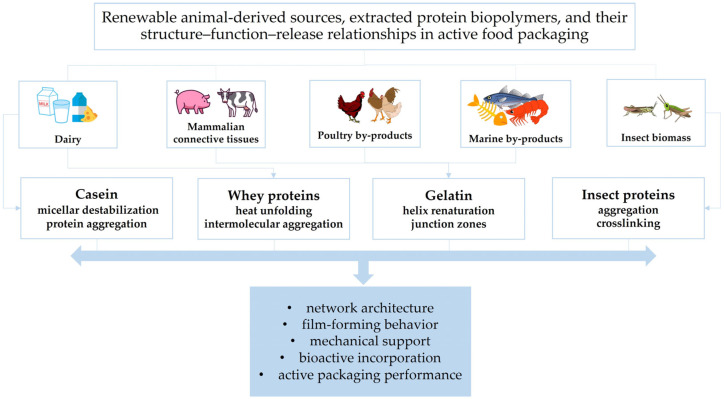
Animal-derived protein biopolymers and their structure–function–release relationships in active food packaging. The arrows indicate the progression from raw material sources to protein types, gelation mechanisms, and the resulting functional properties of the formed gel networks, including network architecture, film-forming behavior, mechanical support, bioactive incorporation, and active packaging performance.

**Figure 2:**
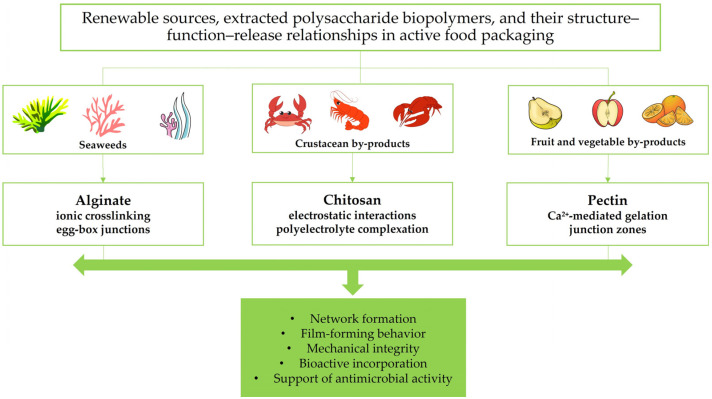
Polysaccharide biopolymers and their structure–function–release relationships in active food packaging. The arrows indicate the progression from renewable sources to polysaccharide types, gelation mechanisms, and final functional properties. The green box represents the resulting functional performance of the formed gel networks, including film-forming ability, mechanical integrity, bioactive incorporation, and antimicrobial activity.

Gel-forming biopolymers are predominantly derived from renewable biological resources of animal, marine, and plant origin and are often obtained from agro-industrial and food-processing by-products, thereby supporting circular bioeconomy strategies and enhancing resource efficiency [50,52,66,67] (Figure 1 and Figure 2).

Figure 1 and Figure 2 illustrate the structure–function–release relationships of the protein- and polysaccharide-based biopolymers considered in this review, linking renewable sources with the dominant gelation-related features that govern their behavior in active food packaging. Taken together, they show how differences in molecular organization and network formation influence bioactive entrapment, diffusion control, packaging performance, and material-specific limitations.

Casein is the major protein fraction in milk, accounting for approximately 80% of total milk proteins, and is widely recognized as an important dairy-derived biopolymer for food applications [68,69]. Its abundance in milk and dairy by-products, including caseinates and cheese whey streams, ensures a continuous and sustainable supply [70,71]. Casein-derived films and hydrogels exhibit favorable biodegradation profiles and represent viable alternatives to conventional plastics [70]. Whey proteins, which account for approximately 20% of milk proteins, are major by-products of cheese, casein, and other coagulated dairy-product manufacturing. They constitute an abundant, renewable, and biodegradable biopolymer source whose intrinsic functional properties make them highly suitable for edible and active packaging materials [30,69]. Gelatin is obtained through partial hydrolysis and thermal denaturation of collagen-rich animal or marine residues, contributing to waste minimization in livestock and fish processing industries [30,39,72]. Owing to its renewability, biodegradability, biocompatibility, cost-effectiveness, and excellent film-forming properties, gelatin is widely used in food packaging [30,73,74,75].

Alginate is mainly extracted from brown algae such as *Macrocystis* and *Sargassum*, as well as from certain bacterial strains, including *Pseudomonas* and *Azotobacter* species [53,76,77]. Chitin and its deacetylated derivative, chitosan, are the second most abundant natural polysaccharides after cellulose and are obtained from crustacean exoskeletons (e.g., shrimp, crabs, and lobsters), fungi (e.g., *Fusarium solani*, *Lentinus edodes*), mushrooms, insects, and other marine organisms [30,39,50,51,52,67,72,78]. Comparative studies have shown that insect-derived chitosan, for example from *Acheta domesticus* and *Gryllodes sigillatus*, can generate films with mechanical and barrier properties comparable to, and in some cases better than, those of crustacean-derived chitosan, highlighting its growing relevance as a sustainable alternative for food packaging. This potential is further supported by its good film-forming ability, biodegradability, and applicability in edible films, antimicrobial coatings, and active packaging systems aimed at extending food shelf life while reducing dependence on marine crustacean sources [53,79,80]. Pectin, a complex plant-derived heteropolysaccharide, is mainly extracted from lignocellulosic biomass and fruit-processing by-products, particularly fruit peels [30,53,81]. Due to its high abundance, water solubility, film-forming ability, flexibility, barrier properties against moisture, oxygen, and aroma compounds, and antioxidant potential, pectin is regarded as a sustainable and environmentally friendly alternative for biodegradable packaging applications [82,83]. The valorization of agro-industrial residues as sources of these polysaccharides further reinforces their sustainability profile and supports circular resource-use strategies. Beyond the renewable origin illustrated in Figure 1 and Figure 2, the gel-forming performance of these biopolymers is strongly governed by their molecular architecture and physicochemical characteristics, as discussed in the following section.

### 2.2. Structural and Physicochemical Determinants Governing Gelation Behavior

The gel-forming behavior of biopolymers is fundamentally determined by their molecular architecture and physicochemical characteristics. To support the interpretation of structure-function relationships, Table 1 summarizes the molecular organization, dominant gelation triggers, and resulting network types of the main protein- and polysaccharide-based biopolymers discussed in this review.

Mechanistically, protein-based gels are generally formed through unfolding and aggregation processes triggered by thermal, pH, or ionic changes, with hydrophobic interactions, hydrogen bonding, electrostatic forces, and, in some systems, disulfide bonds contributing to network formation [30,39,47,50,84,85,86,87,88,89,90,91,92,93,94,95,96,97,98]. In contrast, polysaccharide-based gels are more commonly formed through crosslinking-driven mechanisms, including ionic bridging, hydrogen bonding, electrostatic complexation, and junction-zone formation, depending on polymer type and environmental conditions [39,47,51,52,53,99,100,101,102,103,104]. Thus, protein gelation is predominantly aggregation-driven, whereas polysaccharide gelation is more often crosslinking-driven, leading to distinct structure–function relationships in food packaging matrices [30,39,97,99,101,103].

### 2.3. Limitations of Single-Component Biopolymer Films

Protein gels frequently exhibit aggregation-driven network densification, whereas polysaccharide systems display viscoelastic behavior governed primarily by crosslink density. Although these distinct gelation pathways provide valuable design flexibility, they also impose characteristic performance trade-offs when used as single-component matrices. Protein-based gels are often affected by network heterogeneity, moisture sensitivity, and limited thermal stability, whereas polysaccharide-based gels more commonly exhibit excessive hydrophilicity, ionic sensitivity, and insufficient mechanical robustness under variable conditions [30,39,54,71,72,104,105,106,107]. As a result, mono-component matrices rarely provide the balanced combination of mechanical performance, barrier functionality, and environmental stability required for advanced food packaging applications, which reinforces the relevance of hybrid protein–polysaccharide systems as structurally complementary alternatives [39,51,97]. These constraints highlight the need for structural reinforcement or functional complementation strategies. To provide a structured overview of the intrinsic performance limitations associated with mono-component matrices, Table 2 summarizes the main mechanical, barrier, and environmental constraints of representative protein- and polysaccharide-based biopolymers.

Overall, single-component protein-based films generally offer good film-forming ability and, in some cases, superior mechanical integrity, but they are often highly sensitive to moisture and may exhibit limited barrier stability under humid conditions. In contrast, polysaccharide-based systems are frequently associated with more regular crosslinked networks and, in some cases, favorable gas-barrier properties, yet they often suffer from brittleness, high water affinity, ionic sensitivity, and limited flexibility or dimensional stability. These differences indicate that neither class alone can simultaneously optimize mechanical robustness, moisture resistance, and controlled bioactive retention/release, which helps explain the growing interest in protein–polysaccharide hybrid and ternary systems as more balanced functional matrices [30,33,39,54,71,72,105,106,107]. From a comparative mechanistic perspective, these limitations should not be viewed simply as material-specific disadvantages, but rather as consequences of different network-forming logics. Protein-based systems rely more heavily on aggregation-driven structuring, which can favor film formation and mechanical integrity, but often increase sensitivity to moisture and environmental fluctuations. By contrast, polysaccharide-based systems are more commonly governed by crosslinking-mediated organization, which may promote structural regularity and, in some cases, gas-barrier performance, yet often at the expense of flexibility, water resistance, or dimensional stability. This distinction helps explain why single-component systems rarely achieve balanced multifunctionality and why hybridization has emerged not merely as a compositional strategy, but as a mechanistically grounded design solution.

## 3. Protein–Polysaccharide Hybrid Systems

From a gel mechanics perspective, the inability of single-component matrices to concurrently optimize elasticity, barrier performance, and environmental stability exposes fundamental material constraints. Hybrid protein–polysaccharide systems, therefore, emerge as structural solutions, enabling the integration of aggregation-driven protein gelation with polysaccharide-dominated electrostatic and ionic crosslinking within a unified network architecture [30,39,97]. In many systems, this mechanistic complementarity translates into improved tensile strength, reduced moisture sensitivity, and enhanced structural stability, driven by cooperative intermolecular interactions such as electrostatic attraction, hydrogen bonding, and hydrophobic associations. Importantly, hybridization also creates microenvironments for encapsulation and diffusion-controlled release of antioxidants and antimicrobials, aligning mechanical reinforcement with active functionality in sustainable packaging applications [30,39]. However, the functional superiority of hybrid systems should not be interpreted as universal or formulation-independent. Their performance depends on whether the combination of protein aggregation and polysaccharide-mediated crosslinking generates a network that is sufficiently cohesive to improve strength and barrier performance, yet not so dense that it restricts swelling, bioactive mobility, or diffusion-controlled release. This recurrent trade-off is critical for interpreting the literature, as apparently similar hybrid systems may lead to markedly different outcomes depending on polymer ratio, charge balance, crosslinking density, plasticizer content, and environmental responsiveness. Thus, the key advantage of hybridization lies not in simple component addition, but in the controlled tuning of structure–function relationships.

### 3.1. Gelation Mechanism and Molecular Interactions in Protein–Polysaccharide Systems

**Figure 3:**
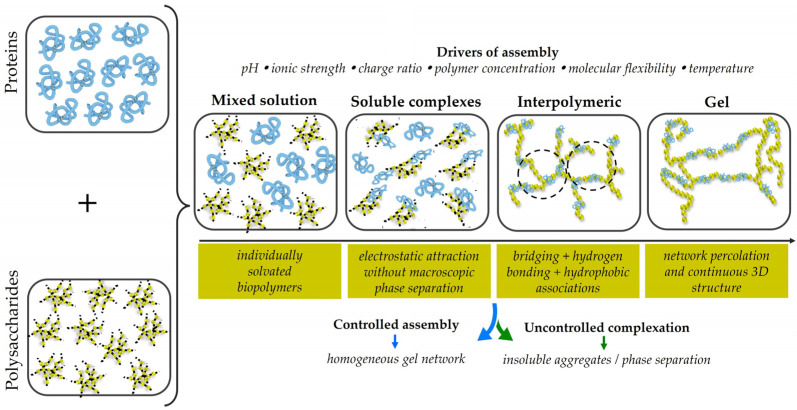
Sequential assembly of binary protein–polysaccharide systems: governing factors, intermolecular organization, and gel network formation. The blue arrow indicates controlled assembly leading to homogeneous gel networks, whereas the green arrow indicates uncontrolled complexation leading to insoluble aggregates or phase separation.(adapted from [32]).

Binary hybrid polymer systems represent the most fundamental class of hybrid materials used in food packaging. These systems can be classified as protein–protein, polysaccharide–polysaccharide, or protein–polysaccharide hybrids. Among them, protein–polysaccharide systems are particularly promising because they combine film-forming ability, mechanical resilience, and barrier functionality [33]. The formation of protein–polysaccharide hybrid networks proceeds through a sequential assembly process governed by physicochemical interactions, as illustrated in Figure 3.

Figure 3 illustrates the progression from individually solvated biopolymers to soluble complexes, interpolymeric associations, and finally a percolated gel network. It also highlights the role of pH, ionic strength, charge ratio, polymer concentration, molecular flexibility, and temperature in directing assembly, as well as the divergence between controlled assembly leading to homogeneous gel formation and uncontrolled complexation resulting in insoluble aggregates or phase separation. Initially, proteins and polysaccharides coexist in a homogeneous mixed solution under suitable pH and ionic strength conditions, where both biopolymers remain individually solvated and no macroscopic structuring is observed. Upon modulation of environmental parameters, electrostatic attraction induces the formation of primary soluble complexes, marking the onset of cooperative assembly without immediate phase separation [32,33]. In the next stage, these complexes progressively organize into more structured supramolecular assemblies, promoted by electrostatic bridging, hydrogen bonding, and hydrophobic interactions that enhance interpolymeric connectivity [33,36,39,55,71]. A similar role of non-covalent interactions has also been reported in recent multicomponent starch-based gel formulations enriched with proteins and hydrocolloids, where hydrogen bonding and electrostatic effects were identified as major contributors to gel strengthening and printability-related performance [43]. Further aggregation of protein–polysaccharide complexes drives structural rearrangement and stabilization, while increasing connectivity ultimately leads to network percolation, the sol–gel transition, and the formation of a continuous three-dimensional structure [32,33]. The final gel architecture depends on charge ratio, molecular flexibility, polymer concentration, aggregation kinetics, and thermodynamic compatibility between the two biopolymers [33].

**Figure 4:**
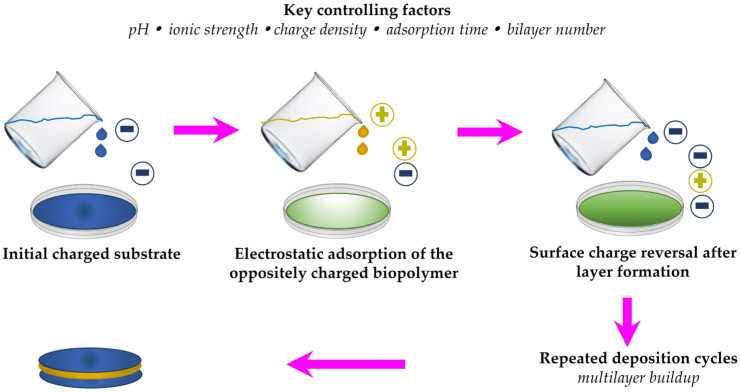
Layer-by-layer self-assembly of protein–polysaccharide multilayers: electrostatic deposition, charge reversal, and interfacial property development. The arrows indicate the sequential deposition process and repeated adsorption cycles leading to multilayer buildup (adapted from [30]).

*Controlled assembly strategy*: Although associative assembly promotes gel network formation, direct blending of oppositely charged proteins and polysaccharides may, under certain conditions, induce excessively strong electrostatic complexation between the two biopolymers. Such uncontrolled interactions can result in insoluble aggregates, phase separation, and heterogeneous structures that compromise film transparency, homogeneity, and mechanical performance. For example, in gelatin/alginate systems, excessively strong electrostatic interactions at non-optimal charge ratios have been reported to induce phase separation or insoluble aggregate formation, thereby disrupting structural uniformity [108]. By contrast, in whey protein/pectin systems, carefully controlled pH conditions favor the formation of soluble complexes or coacervates rather than macroscopic precipitation, illustrating how assembly behavior strongly depends on the balance between charge density and processing conditions [40,62,63,64]. To mitigate these issues and achieve more controlled structural organization, layer-by-layer casting approaches have been introduced (Figure 4) [30,108]. Figure 4 schematically contrasts uncontrolled bulk complexation with sequential electrostatic self-assembly, illustrating why layer-by-layer deposition provides improved control over interfacial organization, multilayer buildup, and structural homogeneity in protein–polysaccharide films.

Unlike direct bulk blending, this technique relies on the sequential deposition of oppositely charged biopolymer solutions, allowing gradual electrostatic assembly at the interface. This strategy minimizes premature aggregation and enables more precise control over film architecture, thickness, and interfacial organization. Layer-by-layer self-assembly involves the alternating adsorption of cationic and anionic polyelectrolytes, repeated as needed to build multilayered structures. The nonspecific nature of electrostatic interactions further facilitates the incorporation of bioactive compounds within the multilayer system, imparting functional properties such as antioxidant and antimicrobial activity. In addition, films fabricated via layer-by-layer self-assembly can provide improved regulation of active compound release into food matrices, thereby enhancing their applicability in controlled-delivery packaging systems [30]. Building on this binary structural scaffold, the incorporation of bioactive compounds transforms protein–polysaccharide gels into ternary hybrid systems capable of functioning as active delivery platforms. In this context, the gel matrix evolves from a structural material into a multifunctional carrier system for antioxidant and antimicrobial delivery in sustainable food packaging.

**Figure 5:**
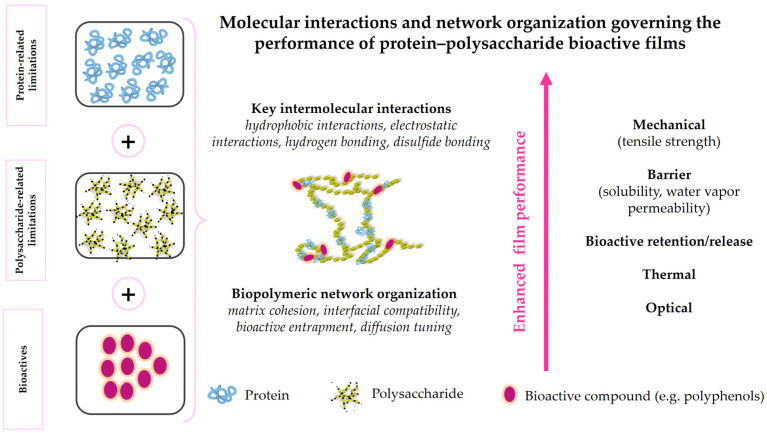
Molecular interactions and network organization governing the performance of protein–polysaccharide films containing bioactive compounds. The arrow indicates the enhancement of film performance resulting from intermolecular interactions and network organization, influencing mechanical, barrier, thermal, optical, and bioactive release properties (adapted from [35]).

Figure 5 provides an integrative overview of the main intermolecular forces involved in ternary protein–polysaccharide–bioactive systems and shows how these interactions are linked to structural reinforcement and functional outcomes such as mechanical stability, barrier performance, and controlled release behavior. Three primary interactions act synergistically to enhance network stability: electrostatic interactions, hydrogen bonding, and hydrophobic associations. Electrostatic interactions between positively charged amino groups and negatively charged carboxyl groups represent a major driving force for complex formation and network cohesion in protein–polysaccharide systems, thereby improving mechanical strength, water resistance, and barrier properties [35,55,71]. The establishment of these interactions depends strongly on pH and on the ionization state of the interacting biopolymers. Proteins may contribute both amino and carboxyl groups depending on their net charge, while anionic polysaccharides such as alginate and pectin mainly provide carboxyl groups. Chitosan is a notable exception due to the presence of protonated amino groups under acidic conditions [71]. Effective electrostatic complexation therefore requires pH conditions under which the interacting biopolymers carry opposite net charges [71]. Hydrogen bonding further stabilizes the interpolymeric matrix and reduces water penetration, solubility, and moisture sensitivity. These interactions may occur between hydroxyl groups of polyphenols and carbonyl or peptide groups of proteins, as well as between polyphenol hydroxyl groups and polysaccharide amino groups. Additional hydrogen bonds may form between carbonyl groups of proteins and amino groups of polysaccharides, further reinforcing the three-dimensional structure. Hydrophobic interactions between the non-polar aromatic rings of polyphenols and hydrophobic amino acid residues of proteins enhance molecular compatibility and promote cohesive network assembly [35,71]. The incorporation of bioactive compounds introduces additional interaction sites, modifying network architecture and influencing functional properties such as solubility, water vapor permeability, tensile strength, and antimicrobial activity. Water-mediated interactions also play a critical role, as the balance between hydrophilic and hydrophobic domains governs barrier performance and matrix flexibility [35]. In addition, additives should enhance functional performance while maintaining film uniformity [71]. Phenolic-rich plant extracts can reinforce the hydrogel matrix through multiple intermolecular interactions, especially hydrogen bonding with amino, amide, and hydroxyl functionalities, thereby contributing to network stabilization at moderate concentrations [35,41,71,109]. However, excessive incorporation may lead to aggregation phenomena or preferential association with one polymer phase, disrupting interpolymeric organization, increase structural heterogeneity, and ultimately impairing film integrity and mechanical performance [30,41,110]. Moreover, chemical crosslinking or disulfide bond formation may further reinforce the polymer network, resulting in enhanced structural integrity and overall film performance [35,39].

### 3.2. Binary Biopolymer Systems

*Casein/chitosan binary system*: The casein/chitosan binary system is governed by the complementary charge characteristics and gelation mechanisms of its components. Casein forms aggregation-driven protein networks with cohesive mechanical behavior, while chitosan provides cationic functionality and electrostatic crosslinking capacity, partially compensating for casein’s moisture sensitivity. Under conditions where casein carries negatively charged carboxylate groups and chitosan remains protonated (–NH_3_^+^), electrostatic complexation promotes the formation of a polyelectrolyte network. Additional stabilization arises from hydrogen bonding between amide, hydroxyl, and carboxyl groups, while hydrophobic interactions enhance further interchain cohesion. Together, these interactions increase the effective crosslink density relative to mono-component films [54,55,56,57,58,70,71]. Hybrid casein/chitosan films typically exhibit smoother and denser microstructures, improved interpolymeric connectivity, enhanced tensile strength, and better barrier performance [55,56]. For example, Xu et al. reported that the tensile strength increased from 10.1 ± 0.37 MPa for neat chitosan film to 11.5 ± 0.4 MPa for the chitosan–casein film, while the incorporation of 2% casein reduced the water vapor permeability of chitosan film by 48%, confirming the reinforcing and barrier-enhancing effect of casein–chitosan hybridization [55]. More recent studies have further supported the relevance of this binary system for food packaging applications by demonstrating promising physicochemical performance in edible hydrogel membranes and identifying blending/crosslinking strategies as effective routes for overcoming the intrinsic moisture sensitivity and limited elasticity of casein-based matrices [38,71]. Nevertheless, both polymers remain inherently hydrophilic, resulting in residual water sensitivity and relatively high water vapor permeability. For this reason, the incorporation of bioactive compounds has been explored as a further strategy to improve stiffness, UV resistance, antioxidant activity, and antimicrobial functionality [55].

*Gelatin/alginate binary system*: The gelatin/alginate binary system is driven by the complementary physicochemical characteristics and gelation pathways of its components. Gelatin forms thermoreversible, aggregation-based physical networks through partial triple-helix renaturation, providing elasticity and cohesive film-forming capacity. However, its intrinsic hydrophilicity results in high water vapor permeability and moisture sensitivity [56,59,97,108]. By contrast, alginate forms ionically crosslinked networks in the presence of divalent cations, contributing structural rigidity and dimensional stability. Hybrid gelatin/alginate systems are mainly governed by electrostatic complexation between positively charged gelatin amino groups (–NH_3_^+^) and negatively charged alginate carboxylate groups (–COO^−^), supplemented by hydrogen bonding [59,60,97]. In Ca^2+^-containing systems, alginate domains additionally undergo egg-box crosslinking, generating localized rigid zones embedded within the more flexible gelatin matrix [99]. This dual-network architecture increases crosslink density and reduces free volume, improving mechanical stability and resistance to deformation under humid conditions. From an application perspective, gelatin–alginate films have been widely explored for biodegradable and edible food packaging due to their balanced combination of flexibility and structural integrity [59,60,108]. These systems are especially suitable for coatings and films for fresh and minimally processed foods, where transparency, film-forming ability, and oxygen barrier properties are desirable [59,108]. Their compatibility with bioactive compounds also enables the development of active packaging materials with antioxidant and antimicrobial functionalities, thereby supporting shelf-life extension and food quality preservation [60,108]. However, excessive strong electrostatic interactions at non-optimal charge ratios may induce phase separation or insoluble aggregate formation, compromising structural homogeneity and film transparency [108].

*Whey proteins/pectin binary system*: The whey protein/pectin system has attracted interest due to the complementary structural and interfacial properties of its components. Whey proteins are widely recognized for their strong film-forming ability, mechanical strength, and gas barrier performance, along with superior emulsifying capacity due to their amphiphilic globular structure [30,61]. However, whey protein-based matrices may exhibit limited long-term structural stability and sensitivity to environmental conditions [39]. Pectin, an anionic heteropolysaccharide with a high degree of esterification, can provide both functionality and steric stabilization under appropriate formulation conditions. In particular, under controlled pH conditions, especially below the isoelectric point of β-lactoglobulin (pI~5.2), positively charged whey proteins can interact with negatively charged pectin carboxylate groups, promoting formation of soluble complexes or coacervates [40,62,63,64,65]. The resulting structural, mechanical, and barrier properties are therefore system-dependent and influenced by pH, biopolymer ratio, ionic environment, and processing conditions. Thermomechanical treatment may further induce protein unfolding, exposing hydrophobic domains and reactive groups that facilitate inter-protein aggregation and electrostatic bridging with pectin. Recent mechanistic studies have shown that such treatment can lead to structurally reorganized complexes in which both protein denaturation and pectin redistribution contribute to the final architecture [37]. From a functional perspective, this reorganization may be advantageous for tailoring matrix cohesion, interfacial behavior, and encapsulation capacity in biodegradable packaging systems [37,61,111]. However, these benefits are not universal and remain highly dependent on formulation variables such as pH, protein–polysaccharide ratio, ionic strength, and processing history [65,111]. In addition, the environmental sensitivity of whey protein-based matrices and their potentially limited long-term structural stability may restrict application under demanding storage conditions [65]. Accordingly, the whey protein/pectin system appears particularly relevant for active packaging applications requiring strong network organization, moderate barrier functionality, and bioactive incorporation [61,102], rather than as a universally superior solution for all packaging contexts. Compared with more structurally robust systems, whey protein/pectin assemblies may offer greater reinforcement through electrostatic complexation but may also show lower environmental robustness and stronger dependence on formulation conditions [65,111]. These assemblies are often interpreted through a core–shell model, in which whey protein-rich domains constitute the core and pectin forms an interfacial shell that contributes steric stabilization and improved colloidal stability [37,39,40,62,63,64,65,112,113,114,115]. This interpretation is supported by studies showing interfacial localization of pectin around protein-rich structures under appropriate pH and thermomechanical conditions [37,65,112,113,114]. At the same time, evidence suggests that partial incorporation of pectin within three-dimensional aggregates may also occur, indicating more complex network architectures [65,114]. Thermomechanical treatment induces protein denaturation and unfolding, exposing hydrophobic domains and reactive groups that enhance inter-protein aggregation and electrostatic bridging with pectin [115].

The incorporation of pectin generally enhances emulsion stability and reduces droplet coalescence through steric and electrostatic stabilization. As a result, hybrid systems may show greater resistance to phase separation than pure whey protein matrices, improved mechanical strength relative to pure pectin films, better moisture regulation than whey proteins alone, and superior emulsion stability for encapsulated lipophilic bioactives such as essential oils or omega-3 fatty acids.

*Comparative rheological perspective of binary systems*. Casein/chitosan, gelatin/alginate, and whey proteins/pectin gels exhibit distinct viscoelastic signatures governed by their dominant interaction mechanisms. Comparative studies indicate that these systems generally display gel-like behavior, commonly characterized by storage modulus (G′) values exceeding loss modulus (G″), although the absolute magnitude of the rheological response varies substantially with biopolymer ratio, pH, ionic strength, thermal history, and oscillatory testing conditions [116,117]. In gelatin/alginate-related film-forming dispersions, rheological behavior has been shown to depend strongly on polymer concentration, with alginate systems displaying consistency coefficient (*k*) values of 0.66, 2.51, and 7.08 Pa·s^n^ at 1.0%, 1.5%, and 2.0%, respectively, together with a decrease in flow behavior index (*n*) from 0.71 to 0.56, reflecting progressively stronger pseudoplasticity and network structuring [118]. By contrast, whey protein/pectin systems tend to display pronounced environmental responsiveness, particularly under pH- and salt-dependent complexation conditions. Raei et al. reported gel-like coacervate behavior with G′ > G″ across the studied systems and identified the strongest viscoelastic response at pH 3.5, while Wang et al. showed that in β-lactoglobulin/pectin coacervates, G′ remained significantly higher than G″ and increased with NaCl concentration up to 0.21 M, followed by a decline at higher salt levels [111,119]. Taken together, these comparisons confirm that the rheological behavior of binary protein–polysaccharide systems is strongly condition-dependent and mechanistically governed by the type of intermolecular association, which ultimately influences their suitability for specific food packaging applications. The binary protein–polysaccharide systems discussed above establish the structural scaffold necessary for mechanical reinforcement and barrier optimization. Their full potential in sustainable packaging applications, however, emerges when these matrices are further functionalized through the incorporation of bioactive compounds. The transition from binary structural networks to ternary functional systems enables the integration of antioxidant and antimicrobial activity within mechanically stable gel matrices.

## 4. Protein–Polysaccharide Ternary Systems for Active Packaging

Binary protein–polysaccharide networks provide the structural basis for mechanical reinforcement and barrier optimization [39]. Their full technological value in food packaging, however, emerges after functionalization with bioactive compounds, which transforms passive matrices into active delivery platforms [30]. In ternary systems, bioactives such as polyphenols, essential oils, and omega-3 fatty acids are incorporated into the three-dimensional network, where they interact with both polymer components and modify crosslink density, mesh size, and network homogeneity [35,55,103,113]. Depending on their concentration and interaction strength, these active agents may either reinforce or plasticize the matrix, thereby altering mechanical performance and permeability [39,55]. From an application standpoint, ternary systems have been widely investigated for active food packaging of perishable products. Protein–polysaccharide films containing plant-derived polyphenols or essential oils have been applied as coatings and active films for fresh fruits, vegetables, meat, and fish products, where they contribute to microbial inhibition, oxidative stabilization, and delayed spoilage [41,108,120]. Likewise, the incorporation of omega-3 fatty acids and other lipid-based bioactives into biopolymer matrices has been explored for functional packaging and controlled-release applications, further highlighting the potential of ternary systems to extend shelf life while maintaining food quality and safety [30,55].

### 4.1. Molecular Interactions Governing Bioactive Incorporation

In ternary systems, molecular interactions are more complex than in binary assemblies because competitive and cooperative associations occur simultaneously among proteins, polysaccharides, and bioactive compounds [41]. Depending on their chemical structure, polarity, and ionization state, polyphenols are among the most extensively investigated antioxidants in protein–polysaccharide matrices because their hydroxylated aromatic structures allow multiple interaction pathways [30,39,41,42,109]. Hydrogen bonding commonly occurs between phenolic hydroxyl groups and protein carbonyl or amide groups, as well as polysaccharide hydroxyl groups. At the same time, hydrophobic interactions between aromatic rings and nonpolar amino acid residues promote molecular association, while pH-dependent ionization may introduce additional electrostatic contributions [41,109]. At moderate concentrations, these interactions can increase effective crosslink density and reduce free volume, thereby improving mechanical cohesion and lowering permeability [29,30]. At higher loadings, however, polyphenols may induce aggregation or preferentially bind to one polymer phase, disrupting protein–polysaccharide complexation and increasing structural heterogeneity [109]. In this sense, polyphenols may act either as molecular bridging agents that reinforce interpolymeric connectivity or as blocking agents that weaken network integrity [41]. As a result, the behavior of ternary systems cannot be directly extrapolated from binary models, and synergistic or antagonistic effects must be experimentally resolved.

Essential oil incorporation follows a different mechanistic route and is governed mainly by emulsion-mediated entrapment rather than specific molecular binding. Amphiphilic proteins stabilize dispersed lipid droplets, while polysaccharides increase viscosity and impose steric constraints, together limiting droplet coalescence and volatilization during film formation and storage [42]. At high concentrations, hydrophobic inclusions may increase free volume and act as plasticizers, thereby reducing intermolecular cohesion and mechanical strength [110]. When essential oils are incorporated as nanoemulsions, however, improved dispersion and matrix-droplet interactions may enhance barrier performance and functional stability [44]. Beyond these formulation effects, essential-oil-loaded films have also shown clear antimicrobial activity against relevant foodborne and spoilage microorganisms. For example, citronella-oil-containing soy protein films inhibited *Staphylococcus aureus* and *Escherichia coli* while improving grape preservation during storage [121]. Oregano-essential-oil-loaded pea protein films similarly suppressed bacterial growth and showed practical potential for poultry packaging applications [46]. In addition, slow-release films containing clove essential oil produced measurable inhibition zones against both *Staphylococcus aureus* and *Escherichia coli* and contributed to bread shelf-life extension, highlighting the importance of sustained release for prolonged antimicrobial functionality [122]. These findings indicate that the technological relevance of essential oils in ternary protein–polysaccharide systems lies not only in their encapsulation and retention within the matrix, but also in their ability to deliver biologically active concentrations capable of suppressing microbial proliferation during storage. In such systems, release is primarily diffusion-controlled through hydrated domains, while network tortuosity and droplet size distribution strongly influence sustained antimicrobial efficacy. More densely interconnected hybrid networks generally promote slower and more sustained migration, whereas looser structures may favor faster initial release.

Long-chain polyunsaturated fatty acids (PUFAs), such as EPA and DHA, introduce additional formulation challenges due to their hydrophobicity and high susceptibility to oxidation. These compounds are typically incorporated in emulsified or microencapsulated forms to improve oxidative stability and modulate release [45,123]. Within hybrid gels, proteins stabilize lipid droplet interfaces, whereas polysaccharides increase matrix rigidity and reduce oxygen diffusion, thereby improving chemical stability [124]. Oxidative stability in omega-3-loaded systems is commonly assessed through primary and secondary lipid oxidation markers during storage. Primary oxidation is usually evaluated by peroxide value, whereas secondary oxidation is frequently monitored by p-anisidine value or thiobarbituric acid reactive substances (TBARS/TBA) [124,125,126]. In more detailed studies, volatile aldehydes and changes in fatty acid composition are also measured to better characterize oxidation progression and PUFA degradation [124,127]. Lipidic inclusions create hydrophobic microdomains that modify network topology; depending on droplet size and loading, they may either reinforce interfacial cohesion or increase heterogeneity. Although release remains predominantly diffusion-driven, oxidative degradation kinetics and matrix hydration state also influence the effective availability of encapsulated PUFAs [128]. Across all bioactive classes, incorporation alters internal organization by modifying crosslink density, mesh size, and polymer mobility, which in turn affects swelling, permeability, and mechanical stability [41,42]. Bioactive incorporation should therefore be regarded not simply as an additive strategy, but as a structural design parameter that directly shapes network organization, encapsulation efficiency, and release behavior in protein–polysaccharide matrices [33,42,129].

### 4.2. Controlled Release Mechanisms in Active Protein–Polysaccharide Packaging Systems

While Section 4.1 addressed the structural incorporation of bioactives into hybrid networks, this section examines how those structural modifications govern mass transfer under realistic food packaging conditions. In active packaging applications, controlled release from protein–polysaccharide gels is intended to maintain sustained antioxidant and antimicrobial activity throughout storage. Because these matrices are exposed to fluctuating humidity, temperature, oxygen levels, and pH, mass transfer behavior is highly condition-dependent [42]. Release from hybrid gels into food or headspace is governed mainly by diffusion through hydrated microdomains, matrix swelling, and environmental stimuli that alter network integrity and polymer mobility [41]. In structurally stable protein–polysaccharide gels, bioactive migration is predominantly diffusion-driven. Transport occurs along concentration gradients through water-filled pores within the crosslinked network, and the effective diffusion rate depends on crosslink density, mesh size, tortuosity, and polymer-bioactive affinity. Densely interconnected hybrid systems generally reduce diffusion coefficients and favor sustained release, which is desirable for prolonged preservation of fresh produce, meat, and dairy products [104]. By contrast, loosely structured matrices may exhibit rapid initial release, as often reported for essential-oil-loaded edible films [42,44]. Quantitatively, release from active films is commonly described using Fickian diffusion models or empirical equations such as the Higuchi and Korsmeyer–Peppas models, depending on matrix structure and the dominant transport mechanism [130,131,132]. For example, gallic acid released from caseinate/guar gum films showed diffusion coefficients ranging from 4.5 × 10^−12^ to 8.1 × 10^−12^ m^2^ s^−1^, with diffusivity decreasing as matrix interactions became stronger [130]. Similarly, diffusion-controlled release in active biopolymer systems is often supported by good fitting to Korsmeyer–Peppas kinetics, particularly in organized matrices designed for sustained release [132]. Polyphenol-loaded polysaccharide films likewise frequently exhibit diffusion-limited antioxidant migration when strong hydrogen bonding occurs between phenolic hydroxyl groups and polymer chains [131]. In hydrophilic matrices, especially those rich in alginate or pectin, water absorption induces polymer relaxation and expansion of the gel network. This swelling increases free volume and enlarges diffusion pathways, thereby accelerating bioactive migration [41]. Under high relative humidity, increased water uptake enhances water vapor permeability, promotes polymer chain mobility, and accelerates the release of incorporated bioactive compounds. Such behavior has been reported in polysaccharide-dominant films exposed to humid storage conditions [42,44]. In packaging practice, swelling-controlled release is particularly relevant for high-moisture foods such as fresh fruits and seafood. Protein–polysaccharide complexes assembled through electrostatic interactions are also inherently sensitive to pH. Variations in food surface pH during storage, such as those associated with fruit ripening or microbial metabolism in meat, may alter charge density and destabilize the electrostatic bridge [40]. As a consequence, pH changes can weaken electrostatic crosslinks, increase swelling, enlarge mesh size, and accelerate release. Whey protein/pectin and casein/chitosan systems, for instance, have shown pH-dependent structural transitions that directly influence bioactive retention and diffusion [37,40]. This responsiveness creates opportunities for active packaging systems capable of releasing functional agents in response to spoilage-related environmental changes. Water activity and temperature further modulate release behavior. Higher water activity enhances hydration and diffusion, while low-moisture environments slow both release and volatilization. Increased temperature raises polymer chain mobility and accelerates diffusion rates. In gelatin-based systems, thermal softening may significantly alter transport pathways by increasing segmental mobility within the protein network [133,134,135]. For omega-3-loaded systems, oxidative stability must also be considered together with diffusion kinetics, since hybrid matrices provide partial oxygen barriers that both reduce lipid degradation and modulate release behavior [45,123]. From a mechanistic and design-oriented standpoint, controlled release cannot be evaluated independently of matrix architecture. In many systems, stronger intermolecular interactions and denser networks improve bioactive retention and structural cohesion, but may also slow the release kinetics or reduce diffusion beyond the desirable functional window. Conversely, matrices with higher swelling capacity may facilitate release, while becoming more vulnerable to moisture-induced instability and weaker barrier performance. The most effective ternary systems are therefore not necessarily those with the strongest interactions, but those that achieve a balanced compromise between retention, release, mechanical stability, and environmental responsiveness under realistic application conditions.

### 4.3. Impact on Mechanical and Barrier Properties

The incorporation of bioactive compounds into protein–polysaccharide gel matrices affects not only release behavior but also mechanical strength, barrier performance, and, in some cases, optical properties. Depending on their molecular structure, concentration, and compatibility with the hybrid network, bioactives may act either as reinforcing agents or as plasticizers, creating a trade-off between structural integrity and functional delivery [41,44]. Bioactive compounds may modify intermolecular interactions within the gel matrix. Hydrophobic essential oils and certain low-molecular-weight phenolics can disrupt polymer-polymer associations by increasing free volume and chain mobility. This often results in reduced tensile strength, higher elongation at break, and increased water vapor permeability. Such plasticization effects have been widely reported in essential-oil-loaded films, particularly in polysaccharide-based active systems, especially at higher oil loadings or when dispersion is poorly stabilized [42,44]. By contrast, polyphenols capable of strong hydrogen bonding or electrostatic interactions may enhance effective crosslink density. At moderate incorporation levels, they can enhance tensile strength, reduce permeability, and improve matrix cohesion. For example, Xu et al. showed that condensed tannin incorporation into chitosan/casein films strengthened intermolecular cohesion and improved both mechanical and barrier properties [55]. Similarly, Amiri Samani et al. reported that grape pomace polyphenols promoted stronger interactions within pectin/protein-based biocomposite films, improving matrix integrity and functionality [63]. These findings indicate that the final mechanical outcome depends strongly on concentration, dispersion quality, and polymer compatibility. An inherent trade-off exists between mechanical stiffness and bioactive mobility. Highly crosslinked networks generally provide greater tensile strength, lower oxygen permeability, and better dimensional stability. At the same time, increased crosslink density typically reduces diffusion coefficients and may limit effective release. In contrast, loosely structured or partially plasticized matrices may favor rapid release but compromise mechanical robustness and barrier performance. Design optimization must therefore balance sufficient structural integrity for packaging handling, appropriate permeability control, and sustained bioactive migration [40]. Beyond mechanical modulation, bioactive incorporation can also improve functional properties. Polyphenols with aromatic conjugated structures absorb UV radiation and may reduce photo-oxidation in packaged foods. Films enriched with plant phenolics have shown enhanced UV-shielding capacity, which improves the oxidative stability of lipid-rich products [41,110]. Essential oils such as thymol and carvacrol, as well as phenolic extracts, also provide antimicrobial activity against foodborne pathogens. When release is controlled by hybrid gels, antimicrobial action can be prolonged compared with direct addition of the active compound [42,44]. In this context, the combination of protein-mediated stabilization and polysaccharide-regulated diffusion contributes to longer-lasting antimicrobial performance in active packaging systems.

### 4.4. Safety and Regulatory Considerations

Beyond structural performance and release functionality, the practical implementation of ternary protein–polysaccharide systems requires rigorous assessment of migration behavior, bioactive stability, and scalability. Although bio-based materials offer clear sustainability advantages, safety assessment and regulatory compliance remain essential for real-world application. Active packaging materials must satisfy food-contact regulations that restrict the migration of substances into food matrices. Migration testing is typically performed under standardized conditions that simulate food type, temperature, and storage duration. In the European Union, food-contact materials are regulated under Regulation (EC) No 1935/2004 [136], Commission Regulation (EU) No 10/2011 [137], and Regulation (EC) No 450/2009 for active and intelligent materials [138]. Together, these frameworks establish requirements for safety and migration in food-contact applications. In the United States, food contact substances are regulated by the FDA through food contact notifications, food additive regulations, or Threshold of Regulation exemptions, depending on the intended use and exposure scenario. FDA assessments consider migration testing, cumulative dietary exposure, and toxicological evidence to determine whether there is a reasonable certainty of no harm under the proposed conditions of use [139]. Excessive burst release may result in migration levels that exceed sensory or regulatory thresholds, particularly in fatty food simulants. Controlled-release design is therefore not only a functional objective, but also a regulatory requirement. At the same time, the functional efficacy of active packaging depends on preserving the chemical stability of incorporated bioactives during storage. Major degradation pathways include oxidation of omega-3 fatty acids and phenolics, volatilization of essential oils, hydrolysis of certain phenolic esters, and photodegradation. Hybrid protein–polysaccharide matrices may improve stability by limiting oxygen diffusion, reducing light transmission through UV-blocking effects, and restricting volatilization via network entrapment. Nevertheless, environmental factors such as humidity and temperature fluctuations can accelerate degradation and shorten the functional lifetime of active compounds [45]. Encapsulation approaches, including nanoemulsions, multilayer systems, microcapsules, and nanofiber-based carriers, have been increasingly used to protect sensitive bioactives against oxidation, volatilization, and premature release. These strategies have been shown to improve the oxidative stability of omega-3-rich compounds, enhance retention of volatile actives, and prolong antimicrobial or antioxidant performance in active packaging systems [45,123,124,128]. Although most current studies rely on laboratory-scale casting and solvent-evaporation methods, industrial implementation requires scalable, cost-effective processing routes. Key considerations include compatibility with extrusion, coating, spraying, and multilayer conversion technologies while maintaining reproducible crosslink density, homogeneous bioactive dispersion, and adequate shelf-life stability of film-forming formulations. Additional constraints include process throughput, drying efficiency, and batch-to-batch reproducibility, all of which directly influence product consistency and regulatory reliability [40,44,45]. Variability in natural raw materials, such as plant-derived polysaccharides or marine proteins, may influence composition, migration behavior, and mechanical performance, making standardization and quality-control protocols essential for industrial implementation [39,47]. Although strategies such as layer-by-layer self-assembly and nanoemulsion incorporation are effective at the laboratory scale, they may pose challenges in terms of industrial throughput and process control [40]. Ultimately, successful industrial adoption will depend on balancing performance enhancement, regulatory compliance, cost efficiency, and environmental impact.

To synthesize the comparative structure–function–application relationships discussed throughout this review, Table 3 presents an integrative framework linking dominant intermolecular interactions and network organization to mechanical performance, barrier properties, and controlled release behavior across protein-based, polysaccharide-based, hybrid, and ternary gel systems.

The table summarizes how dominant intermolecular interactions determine network organization and, consequently, mechanical performance, barrier properties, and controlled-release behavior, while also highlighting the main limitations and typical applications associated with each system type. To provide a comparative overview of structure–function relationships across different biopolymer system types, Table 4 presents a semi-quantitative comparison of mechanical properties, barrier performance, structural stability, controlled release behavior, and formulation complexity for protein-based, polysaccharide-based, hybrid, and ternary systems. This comparison highlights the main functional trade-offs associated with each system type and supports the structure–function–application framework discussed throughout this review.

As shown in Table 4, single-component protein and polysaccharide systems present complementary advantages but also intrinsic limitations in terms of moisture resistance, mechanical strength, or controlled release performance. Hybrid systems partially overcome these limitations by combining aggregation-driven protein networks with polysaccharide-based electrostatic or ionic crosslinking, resulting in improved structural stability and more balanced barrier properties. Ternary systems, in which bioactive compounds are incorporated as a third functional component, provide the highest potential for controlled release and multifunctional performance, but also introduce greater formulation complexity and stability challenges. These comparative trends support the idea that bioactive incorporation should be considered a structural design parameter rather than a simple additive strategy, reinforcing the need for an integrated structure–function approach in the design of active packaging materials.

## 5. Limitations, Challenges, and Future Perspectives

Beyond the system-specific trends summarized in Table 3 and Table 4, several broader scientific, technological, and translational challenges still limit the predictability, reproducibility, and practical implementation of protein–polysaccharide gel carriers for active food packaging. At the material level, these systems remain highly sensitive to formulation-dependent interactions, crosslink density, and environmental conditions, often leading to heterogeneous network organization, variable moisture resistance, and inconsistent mechanical or barrier performance. These limitations reflect the intrinsic trade-offs of biopolymer-based matrices and become even more pronounced in hybrid and ternary assemblies, where incomplete component compatibility may promote localized aggregation, phase heterogeneity, or weak interfacial cohesion depending on polymer ratio, charge balance, processing history, and bioactive incorporation [29,30,33,39,54,71,72,105,106,107].

A major challenge lies in the predictive control of structure–function–release relationships in ternary systems, where competitive and cooperative molecular interactions alter crosslink density, mesh size, and diffusion pathways in a non-linear manner [41,42]. Stronger intermolecular interactions and denser networks may improve matrix cohesion, tensile resistance, and barrier performance, but they may also reduce diffusion coefficients and delay release kinetics beyond the desirable functional range. Conversely, loosely structured or highly swollen matrices may facilitate faster bioactive migration while compromising mechanical integrity and barrier stability. These trade-offs indicate that optimal systems are not necessarily those with the strongest interactions, but those able to achieve a balanced compromise between retention, release, structural stability, and environmental responsiveness under realistic application conditions [33,40,41,42,43,44,104,130,131,132]. Future research should therefore place greater emphasis on quantitative structure-release models validated under realistic packaging scenarios, including variable humidity, temperature, food simulants, and extended shelf-life conditions.

Another persistent limitation is the low comparability of published results. Apparently similar formulations are often evaluated under different processing conditions, storage environments, film thicknesses, plasticizer contents, drying methods, relative humidity levels, and analytical protocols, making cross-study interpretation and quantitative benchmarking difficult [40,44,45]. As a result, transferable design rules remain poorly defined. This problem is even more pronounced in systems based on agro-industrial or marine by-products, where compositional variability may directly affect safety, reproducibility, migration behavior, and mechanical consistency [39,40,44,45,47]. Greater standardization of raw materials, testing strategies, and reporting practices is therefore required.

An important future direction is the development of stimuli-responsive systems capable of modulating bioactive release in response to pH, humidity, temperature, oxidation state, or microbial activity [39,40]. However, the challenge lies not only in achieving responsiveness, but also in ensuring selective triggering, reproducible performance, and preservation of mechanical integrity during storage and handling. Computational modeling and AI-assisted material design may further accelerate formulation optimization, although their practical value will depend on larger experimental datasets, harmonized descriptors, and better standardized input variables [30,42]. In this context, recent multicomponent hydrogel studies developed outside the food-packaging field suggest that hierarchical network engineering and data-driven rheological modeling may provide useful conceptual guidance for the next generation of smart active packaging matrices. More broadly, recent hybrid hydrogel systems developed outside the food-packaging field show that hierarchical assembly between polymeric and inorganic components can significantly influence gelation behavior, viscoelastic stability, and sustained-release performance, underscoring the broader relevance of network engineering for multifunctional gel systems [140].

From an industrial perspective, broader implementation remains limited by processability, scale-up requirements, and regulatory validation. Although casting and solvent-evaporation methods still dominate the literature, industrial implementation requires compatibility with scalable technologies such as extrusion, coating, spraying, and multilayer processing [40,44,45]. At the same time, batch-to-batch reproducibility, bioactive dispersion uniformity, long-term storage stability, and the effects of sterilization, drying, and packaging-line conditions on gel structure and release behavior remain insufficiently explored. Regulatory assessment is equally critical, since active packaging systems must comply with food-contact frameworks governing migration limits and safety under intended conditions of use [136,137,138,139]. Additional difficulties arise when active compounds are highly sensitive to oxidation, volatilization, hydrolysis, or photodegradation, requiring stabilization strategies that remain compatible with both processability and safety requirements [123,124,128].

Overall, future research should move beyond formulation screening and adopt a more integrated strategy linking molecular-level design, predictive release engineering, scalable processing, and regulatory validation. Progress in these areas will be essential to translate protein–polysaccharide gel carriers from promising laboratory systems into industrially relevant next-generation active food packaging technologies [40,44,45].

## 6. Conclusions

Protein–polysaccharide gel systems represent a versatile and sustainable platform for active food packaging. This review shows that gelation mechanisms, crosslink density, and network topology are key determinants of mechanical performance, barrier behavior, and controlled bioactive release. In hybrid systems, the combination of aggregation-driven protein networks with electrostatically crosslinked polysaccharides improves structural stability and enables more controlled diffusion of antioxidant and antimicrobial compounds. In ternary systems, bioactive incorporation further modifies network organization through competitive and cooperative molecular interactions, directly affecting mesh size, permeability, mechanical properties, and release behavior. Controlled release is predominantly diffusion-driven but remains highly sensitive to pH, humidity, temperature, and matrix architecture. As a result, achieving an effective balance between structural integrity and sustained bioactive delivery remains a critical design challenge. Future progress will depend on improved predictive understanding of structure-function relationships, the development of stimuli-responsive and smart gel systems, the integration of AI-assisted formulation strategies, and closer alignment with circular bioeconomy principles. The main contribution of this review is the integration of molecular interactions, network architecture, and functional performance into a comparative structure–function–application framework for protein–polysaccharide gel carriers, with particular emphasis on ternary systems and controlled release mechanisms. By highlighting major trade-offs, current limitations, and scale-up challenges, this review supports a more rational and design-oriented development of next-generation active food packaging materials.

## Figures and Tables

**Table 1:** Molecular architectures, dominant gelation triggers, and resulting network types of selected protein- and polysaccharide-based biopolymers.

Gel-Forming Biopolymers Used in Food Packaging		
Protein-based biopolymers **Predominantly aggregation-driven systems**		
Casein	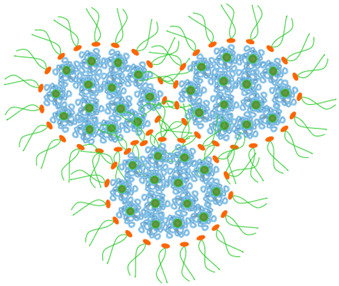 Adapted from [84,85]	Dynamic micellar protein aggregates stabilized by hydrophobic interactions and calcium phosphate nanoclusters; gelation triggered by micelle destabilization induced by acidification, Ca^2+^ addition, enzymatic cleavage, or heating; aggregation-driven formation of a three-dimensional protein network [86,87,88,89,90,91].
Whey proteins	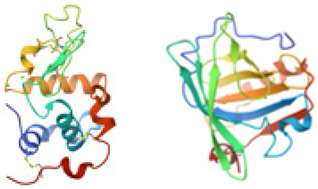 [92,93]	Globular proteins composed mainly of β-lactoglobulin and α-lactalbumin; heat-induced unfolding of β-lactoglobulin (>70–80 °C); disulfide bonding and hydrophobic interactions forming a 3D protein network [30,94,95].
Gelatin	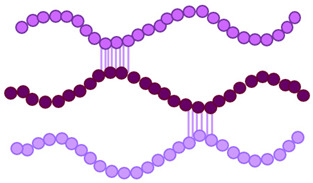 Adapted from [96]	Linear collagen-derived polypeptides with amphiphilic structure and strong film-forming ability; gelation occurs upon cooling through formation of triple-helix junction zones stabilized by hydrogen bonding; triple-helix junction zones forming a thermoreversible physical network [30,39,72,96,97,98].
Polysaccharide-based biopolymers **Predominantly crosslinking-driven systems**		
Alginate	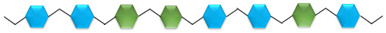 Adapted from [47,99]	Linear anionic polysaccharide composed of mannuronic (M) and guluronic (G) acid blocks; gelation triggered by divalent cations (Ca^2+^) through ionotropic crosslinking; egg-box three-dimensional gel network [39,47,51,53,99,100].
Chitosan	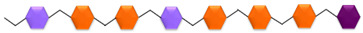 Adapted from [47,101]	Cationic polysaccharide derived from chitin with high charge density; gelation induced by pH reduction or by interaction with polyanions; electrostatic complexation network [30,42,51,53,54,72,78,101,102].
Pectin	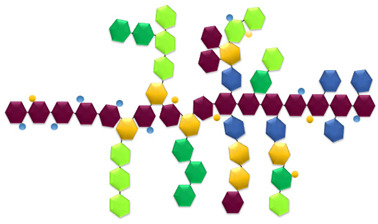 Adapted from [47,103]	Anionic heteropolysaccharide rich in galacturonic acid residues; gelation induced by low pH/high sugar concentrations in high-methoxyl pectin or by Ca^2+^ crosslinking in low-methoxyl pectin; ionic or hydrogen-bonded polysaccharide network [30,47,51,53,103,104].

**Table 2:** Key limitations of selected protein- and polysaccharide-based biopolymers.

Biopolymer	Key Limitations	Refs.
Casein	Limited tensile strength; moisture sensitivity; moderate thermal instability.	[71]
Whey proteins	Moderate mechanical performance; limited moisture barrier capacity; hydrophilicity-induced plasticization; humidity-dependent brittleness; enzymatic degradability.	[30,105]
Gelatin	Low mechanical strength; high water vapor permeability; hygroscopic swelling, limited thermal resistance.	[30,39,54,72]
Alginate	Limited mechanical strength and elasticity; excessive hydrophilicity; ionic sensitivity; poor dimensional stability without crosslinking.	[106,107]
Chitosan	Moderate mechanical properties; humidity sensitivity; limited thermoplasticity; scalability challenges.	[29,30,39,53,54,78]
Pectin	Low tensile strength; high water solubility, pronounced moisture sensitivity; Ca^2+^-dependent mechanical reinforcement.	[39,53]

**Table 3:** Comparative structure–function–application framework for protein-based, polysaccharide-based, hybrid, and ternary gel systems used in active food packaging.

System Type	Dominant Interactions	Network Structure	Mechanical Properties	Barrier Properties	Controlled Release	Main Limitations	Typical Applications
Protein-based systems	Hydrogen bonding, hydrophobic interactions, disulfide bonds	Aggregation-driven, heterogeneous network	Moderate strength, good flexibility	Moderate oxygen barrier, weak moisture resistance	Moderate retention, diffusion often structure-dependent	Moisture sensitivity, thermal instability, structural heterogeneity	Edible films, antioxidant carriers, lipid oxidation control
Polysaccharide-based systems	Ionic crosslinking, hydrogen bonding	Crosslinking-driven, more ordered	Low to moderate strength, brittle behavior	Good oxygen barrier, poor moisture barrier	Diffusion-controlled release, often faster in humid conditions	High hydrophilicity, ionic sensitivity, low flexibility	Oxygen barrier films, antimicrobial coatings
Protein–polysaccharide hybrid systems	Electrostatic interactions, hydrogen bonding, hydrophobic associations	Interpenetrating or complex coacervate network	Improved strength and structural stability	Improved barrier properties compared to single-component systems	More controlled release due to denser network	Compatibility issues, phase separation	Active films, multifunctional coatings
Ternary systems (protein–polysaccharide–bioactive)	Combined electrostatic, hydrogen bonding, hydrophobic interactions between matrix and bioactive	Multi-network structure with bioactive entrapment and diffusion pathways	Good mechanical stability depending on crosslink density	Balanced oxygen and moisture barrier	Controlled and sustained release possible	Formulation complexity, release optimization	Active packaging, antioxidant and antimicrobial delivery systems

**Table 4:** Semi-quantitative comparison of structure–function performance across protein, polysaccharide, hybrid, and ternary biopolymer systems.

Property	Protein	Polysaccharide	Hybrid	Ternary
Mechanical strength	++	+	+++	+++
Flexibility	++	+	++	++
Oxygen barrier	+	+++	++	++
Moisture resistance	+	+	++	++
Structural stability	+	++	+++	+++
Controlled release	+	++	++	+++
Formulation complexity	+	+	++	+++

Note: + low; ++ moderate; +++ high.

## Data Availability

No new data were created or analyzed in this study. Data sharing is not applicable to this article.
